# Chemical Biology Screening Identifies a Vulnerability to Checkpoint Kinase Inhibitors in TSC2-Deficient Renal Angiomyolipomas

**DOI:** 10.3389/fonc.2022.852859

**Published:** 2022-03-10

**Authors:** Robert M. Vaughan, Jennifer J. Kordich, Chun-Yuan Chan, Nanda K. Sasi, Stephanie L. Celano, Kellie A. Sisson, Megan Van Baren, Matthew G. Kortus, Dean J. Aguiar, Katie R. Martin, Jeffrey P. MacKeigan

**Affiliations:** ^1^ Pediatrics and Human Development, College of Human Medicine, Michigan State University, Grand Rapids, MI, United States; ^2^ Center for Cancer & Cell Biology, Van Andel Research Institute, Grand Rapids, MI, United States; ^3^ Graduate Program in Genetics, Michigan State University, East Lansing, MI, United States; ^4^ Obstetrics, Gynecology, and Reproductive Biology, College of Human Medicine, Michigan State University, Grand Rapids, MI, United States; ^5^ Preclinical Research, Tuberous Sclerosis Complex (TSC) Alliance, Silver Springs, MD, United States

**Keywords:** Chk1/2, CHEK1/2, TSC2, tuberous sclerosis complex, mTOR, checkpoint kinase inhibitors, AZD7762, tumor xenografts

## Abstract

The tuberous sclerosis complex (TSC) is a rare genetic syndrome and multisystem disease resulting in tumor formation in major organs. A molecular hallmark of TSC is a dysregulation of the mammalian target of rapamycin (mTOR) through loss-of-function mutations in either tumor suppressor *TSC1* or *TSC2*. Here, we sought to identify drug vulnerabilities conferred by TSC2 tumor-suppressor loss through cell-based chemical biology screening. Our small-molecule chemical screens reveal a sensitivity to inhibitors of checkpoint kinase 1/2 (CHK1/2), regulators of cell cycle, and DNA damage response, in both *in vitro* and *in vivo* models of TSC2-deficient renal angiomyolipoma (RA) tumors. Further, we performed transcriptional profiling on TSC2-deficient RA cell models and discovered that these recapitulate some of the features from TSC patient kidney tumors compared to normal kidneys. Taken together, our study provides a connection between mTOR-dependent tumor growth and CHK1/2, highlighting the importance of CHK1/2 inhibition as a potential antitumor strategy in TSC2-deficient tumors.

## Introduction

The tuberous sclerosis complex (TSC) is a multisystem disease genetically characterized by a loss of function in either of the two tumor suppressors, *TSC1* (hamartin) or *TSC2* (tuberin) (1, 2). We recently used comprehensive genomic profiling of TSC patient tumors and found that ~85% carried mutant *TSC2*, ~12% had mutant *TSC1*, and ~3% had no identified mutation in either *TSC* gene (3). Loss of TSC1 or TSC2 results in benign tumor formation in various organs (4), including subependymal nodules (SEN), subependymal giant cell astrocytomas (SEGA) and cortical tubers of the brain, smooth muscle tissue and cystic changes in the lungs (lymphangioleiomyomatosis, LAM), skin fibromas and angiofibromas, and cardiac rhabdomyomas in infants (5, 6). The kidney is the most common location for lesions in TSC patients, with up to 80% of patients developing renal cysts and angiomyolipoma (RA). RA tumors are associated with spontaneous hemorrhage and require lifelong surveillance (7). Further, the multisystem and numerous organ manifestations can be associated with severe morbidity and potentially death with kidney disease as the highest cause of mortality in TSC patients (8).

Loss of TSC1 or TSC2 leads to constitutive activation of the mammalian target of rapamycin (mTOR) (9), a master regulator of nutrient and energy status in cells. This permits aberrant cell division and growth. Accordingly, TSC tumors show dramatic therapeutic sensitivity to rapamycin (sirolimus) or other rapamycin analogs (rapalogs). However, while sirolimus is effective against various TSC-based tumor lesions, this allosteric mTOR inhibitor is primarily cytostatic, and tumors regrow upon cessation of therapy (10, 11). Furthermore, rapamycin treatment in patients is associated with a decrease in angiomyolipoma volume of ~50%, and unfortunately, these benefits are reversed after treatment is withdrawn (10, 12, 13). However, rapamycin side effects are mild to moderate, and after continuous treatment for 3 years, efficacy was maintained without new or additional significant side effects (14). Due to this cytostatic effect and favorable safety profile, most patients may require a lifelong treatment regimen of rapamycin (15). Therefore, identifying additional therapeutic options for TSC patients that would sensitize TSC2-deficient angiomyolipoma cells might be preferable over lifelong therapy. Here, we sought to identify new TSC2-dependent vulnerabilities using chemical biology approaches and validation in mouse models.

## Materials and Methods

### Cell Culture

621-102 (TSC2-deficient) and 621-103 (TSC2-rescued) were previously generated (16) by stable expression of either an empty vector or *TSC2* in the E6/E7 and hTERT immortalized renal angiomyolipoma 621-101 cells (17). 621-102 and 621-103 cells were maintained in DMEM with 10% FBS. UMB1949 cells were originally isolated from a renal angiomyolipoma and immortalized *via* SV40 large T antigen and hTERT introduction (18). 105K cells were derived from a renal tumor from a *Tsc2* ^+/-^ mouse (19). 621-102, 621-103, and UMB1949 were purchased from ATCC and maintained in DMEM (high glucose) with 10% FBS and 250 U/ml penicillin–streptomycin at 37°C with 5% CO_2_. 105K cells were maintained in DMEM with 10% FBS and penicillin (100 U/ml) and streptomycin (100 µg/ml). For nutrient depletion experiments, cells were plated in complete media (10% FBS). The next day, cells were washed once with PBS and then starved overnight in media with either full media (10% FBS, with nutrients) or starvation media (0.1% FBS, without nutrients).

### Reagents and Antibodies

LY2603618, AZD7762, and rapamycin were purchased from Selleck Chemicals. Antibodies used in Western blotting were TSC2 (Cell Signaling Technologies [CST] #4308), pS6K-T389 (CST #9205), pS6-S235/236 (CST #4858), α-tubulin (CST #2144), CHK1 (CST #2360), pCHK1-S296 (CST #90178), and pCHK1-S345 (CST #2348).

### Chemical Compound Screens

Cell viability assays using a luminescent CellTiter-Glo (Promega) assay were optimized to achieve at least two population doublings in 384-well plates after plating in full media conditions; the result was 700 cells/well and growth time of 72 h for 621-102/621-103. Each of the 384-well screening plates contained positive control compounds, an allosteric mTOR inhibitor (50 nM rapamycin), and cell death control (1 µM staurosporine). All results presented as viability relative to vehicle (DMSO-treated) cells on a per plate basis. The primary screen (621-102 vs. 621-103) included 480 compounds (selected from SelleckChem L1100) at six different concentrations (**Supplemental Table 1**). Validation dose–response curves were generated at 72 h. For the secondary screen (**Supplemental Table 2**), 458 compounds from the primary screen passed quality control and were screened against 621-102 in our optimized growth conditions (DMEM + 0.1% FBS) where 621-103 would not grow (reflective of functional TSC2). The top compounds for relative viability reduction in 621-102 were included (n = 88) in the follow-up screen in UMB1949 and 621-102 cells in 0.1% FBS growth conditions. With the 88 compounds, we performed 10-point dose–response curves in both TSC2-deficient cell lines and calculated the EC_50_ values on the CellTiter-Glo data (**Supplemental Table 3**). The EC_50_ values were calculated in GraphPad Prism using the non-linear fit of [inhibitor] vs. response (three parameters), and the best-fit data are presented.

### siRNA Knockdown

For siRNA knockdown, 2,500 cells were plated in 96-well plates and treated with 25 nM siRNA from Qiagen (AllStars Negative Control, Qiagen SI03650318; AllStars Cell Death Control, Qiagen SI04381048; CHEK1 equimolar pool of SI02660007, SI00299859, SI00605094, SI00287658; or CHEK2 equimolar pool of SI02224271, SI02655422, SI02224264) in siLentFect (Bio-Rad, Hercules, CA, USA) and CellTiter-Glo (CTG, Promega, Madison, WI, USA) was performed 72 h later. Negative and positive controls, including transfection controls, were used to determine Relative Cell Viability (%) with CHK1 and CHK2 knockdown.

### UMB1949 Cell Line Tumorgraft Models

All animal studies were performed in accordance with recommendations of the AAALAC and received institutional IACUC approvals. Prior to establishing cell line tumorgraft models, UMB1949 cells were found negative for mouse hepatitis virus, mouse parvovirus, minute virus of mice, Theiler’s murine encephalomyelitis virus GDVII, *M. pulmonis*, and *mycoplasma* (IDEXX BioResearch, Westbrook, ME, USA). UMB1949 cells (5 × 10^6^) were subcutaneously injected into the right flank of female NSG (NOD *scid* gamma) mice until tumors formed, at which point mice were euthanized and tumors aseptically harvested. The resected tumors were then subdivided to allot material for both cryopreservation and subsequent propagation *in vivo* (<3 mm in size). Tumor specimens were placed into transfer media [RPMI 1640 media (Invitrogen, Carlsbad, CA, USA), 10% fetal bovine serum (Mediatech, Manassas, VA, USA), 1% penicillin/streptomycin (Invitrogen), and 50 units/ml heparin (Sigma, St. Louis, MO, USA)]. Tumor specimens were moved into individual petri dishes of sterile phosphate-buffered saline (Invitrogen, Carlsbad, CA, USA) and separated into <3-mm fragments. Each mouse was treated with the analgesic ketoprofen (5 mg/kg body weight) with betadine (Purdue Products LP, Stamford, CO, USA) being used to sterilize the right flank prior to surgery. While under isoflurane anesthesia, a subcutaneous pocket was subsequently created, and the tumor fragment was inserted prior to closing with surgical staples. Postoperative care included daily animal monitoring for overall health and tumor growth. Tumor volumes were measured by calipers in three dimensions and calculated using the following equation: (½ × length × depth × height). Measurements were taken once weekly when tumor volumes ≤ 100 mm^3^ and three times weekly when > 100 mm^3^. In parallel with these measurements, weekly body weights were also recorded. Treatments were initiated when tumorgraft volume was 400 ± 25 mm^3^. AZD7762 was dissolved (5 mg/ml) in vehicle (11.3% 2-hydroxypropyl-b-cyclodextrin in PBS) and diluted (1 mg/ml) prior to injection at 12.5 mg/kg. For sacrifice, mice were anesthetized with i.p. injection overdose of avertin, followed by perfusion with 10 ml of PBS and removal of subcutaneous tumor into either 4% paraformaldehyde followed by washes in increasing concentration of ethanol to a final of 70%, or into isopentane on dry ice for freezing and long-term storage.

### 105K Xenografts

Seven- to eight-week-old female BALB/c nude mice (Janvier Labs, Le Genest-Saint-Isle, France) were injected subcutaneously into the right flank with 2 × 10^6^ 105K *Tsc2* null cells (19) in 150 µl of DMEM/Matrigel (1:1) by Porsolt SAS (Le Genest-Saint-Isle, France). When the tumors reached 100 mm^3^, treatment group mice (n = 14) were administered either vehicle (2% ethanol, 5% Tween-80, 5% PEG400 in PBS) or AZD7762 (12.5 kg/kg, 1×/day) *via* intraperitoneal injection for 28 days total.

### Cystadenoma Mouse Model

A/J *Tsc2* ^+/-^ mice (20) were maintained through the TSC Alliance Preclinical Consortium by the Van Andel Research Institute. Groups (n = 10) of 8-month-old mice (five male and five female) were treated for 28 days with either AZD7762 or vehicle (12.5 mg/kg, 1×/day, in 5% PEG400, 5% Tween-80, 2% ethanol in PBS). After 28 days, animals were euthanized, and both kidneys were collected for histology. Kidneys were embedded in paraffin, split parasagittal, and serial 5 micron-thick sections were obtained 100 microns apart. Slides were processed on the Discovery Ultra platform (Ventana, Oro Valley, AZ, USA) and imaged using the ScanScope XT digital pathology slide scanner (Aperio, Sausalito, CA, USA) at ×20 magnification. Histological analysis was performed by PsychoGenics Inc. (Paramus, NJ). Dystrophic areas were manually outlined, and the cell content was measured by Image-Pro Premier (v3.2). Lesions were classified as cysts (0%–25%) or cystadenomas (25%–90%).

### Immunoblotting

Cells were lysed in ice-cold lysis buffer (10 mM KPO_4_, 1 mM EDTA, 5 mM EGTA, 10 mM MgCl_2_, 25 mM beta-glycerolphosphate, 50 mM NaF, 1 mM Na_3_VO_4_, 0.5% NP40, 0.1% Brij35, 0.1% sodium deoxycholate, 1 mM DTT, and 1× protease inhibitors (Sigma)). Tumor lysates were prepared by resuspending the pellets in RIPA buffer (150 mM NaCl, 1% NP-40, 0.5% sodium deoxycholate, 0.1% SDS, 50 mM Tris–HCl, pH 8) containing protease inhibitors (100 μM PMSF, 1 mM benzamidine, 2.5 μg/ml pepstatin A, 10 μg/ml leupeptin, and 10 μg/ml aprotinin) and phosphatase inhibitors (1 mM each of NaF, Na_3_VO_4_, and Na_2_P_2_O_7_). Equal amounts of proteins were subjected to SDS-PAGE and transferred to nitrocellulose membranes. Membranes were blocked overnight at 4°C with 5% nonfat milk in TBS-T, followed by incubation in primary and secondary antibodies (1 h at RT, 2% milk in TBS-T). Proteins were detected by enhanced chemiluminescence.

### RNA Isolation

The specific method for RNA isolations was indicated in Martin et al. (3). DNA and RNA were simultaneously isolated using a modified version of the method described in Pena-Llopis and Brugarolas (21). Tissues were lysed and homogenized using mirVana kit lysis buffer (Ambion), a micropestle, and QIAshredder columns (Qiagen, Hilden, Germany). DNA was isolated (targeted TSC2 sequencing) using AllPrep columns (Qiagen), while flow-throughs were used to isolate RNA using an acid phenol–chloroform extraction and the mirVana kit (Ambion). RNA integrity was confirmed using a Bioanalyzer 2100 (Agilent, Santa Clara, CA, USA). RNA concentrations were determined using a Qubit 2.0 fluorometer (Invitrogen).

### RNA Sequencing and Differential Gene Expression Analysis

RNA sequencing of the UMB1949 and 621-102 cell lines was performed under identical published methods of our previous study (3). For RNA sequencing (GSE #189969), polyA-enriched libraries were sequenced with 100-bp paired end reads, aligned to hg19 genome build, and normalized to counts per million (CPM) (**Supplemental Table 3**). The patient RA tumor samples and normal kidney were from Martin et al. (2017) (3). For differential gene expression (DEG) analysis, mitochondrial genes were excluded; the remaining genes were ranked (high to low) by the absolute difference in log_2_ CPM between the average of the normal kidneys and the average of the RA tumors. CIBERSORT was performed as previously described (3), using the latest version 1.05 (3).

### Statistical Analyses

Data are presented as mean ± standard error of the mean (s.e.m.) or mean ± standard deviation (s.d.), as indicated. For all animal models, data are presented as mean ± 95% confidence interval. The EC_50_ calculations represent the best-fit data after fitting to a three-parameter dose–response curve in GraphPad Prism. A one-way ANOVA was performed to measure differences of the histological lesion types.

## Results and Discussion

**Figure 1:**
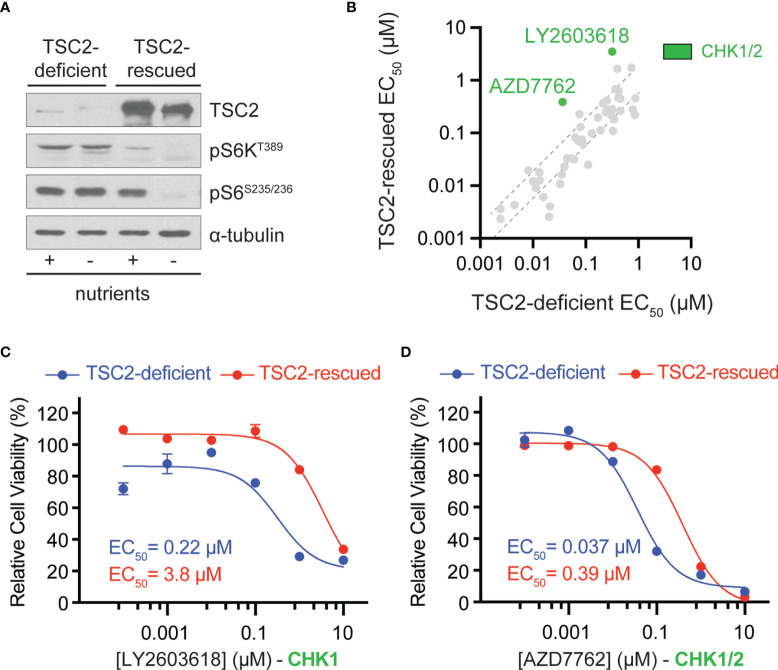
Chemical compound screen identifies sensitivity to checkpoint kinase inhibition in TSC2-deficient cells. **(A)** Western blots from TSC2-deficient (621-102) or TSC2-rescued (621-103) cells cultured with (+) or without (-) nutrients. **(B)** Scatter plot of EC_50_ values after chemical compound screens in TSC2-deficient (x-axis) vs. TSC2-rescued (y-axis) cells with >60% reduction in viability and an EC_50_ value <1 µM in TSC2-deficient cells; dashed black lines represent the 99% confidence interval for the linear regression. CHK1/2, checkpoint kinase 1/2, inhibitors (green). **(C, D)** Dose–response curves for the indicated CHK inhibitors LY2603628 **(C)** and AZD7762 **(D)** in TSC2-deficient (blue) or TSC2-rescued cells (red) after 72 h; data are presented as mean ± s.e.m (n = 3).

To identify therapeutic vulnerabilities in TSC2-deficient tumors, we used a pair of isogenic cell lines derived from a renal angiomyolipoma (RA) cell line 621-101 that were either TSC2-deficient (621-102, control) or TSC2-rescued (621-103, *TSC2* expression) (**Figure 1A**) (16). First, we performed chemical compound screens in each cell line to identify compounds that compromised cell viability greater in the TSC2-deficient setting when compared to the TSC2-rescued cells (**Figure 1** and **Supplemental Table 1**). We used a molecularly targeted library consisting of 480 compounds from a collection of diverse, active, cell-permeable small-molecule inhibitors from preclinical research and clinical trials, including kinase inhibitors, natural products, and chemotherapeutic agents, screened at six concentrations (0.1 nM to 10 µM) for 72-h treatments (**Supplemental Table 1**). Next, we generated six-point dose–response curves for each molecularly targeted compound to generate cellular half maximal effective concentration (EC_50_) values and prioritized compounds that were effective (reduced cell viability > 60% at any concentration) and particularly potent in TSC2-deficient cells (EC_50_ < 1 µM). By plotting EC_50_ values in TSC2-deficient versus TSC2-rescued cells (**Figure 1B**), we identified two compounds targeting the serine/threonine-protein checkpoint kinase 1 and 2 (CHK1 and CHK2), LY2603618 and AZD7762, as selective for TSC2-deficient cells, which were selected for additional interrogation. AZD7762 is equally potent against CHK1 and CHK2, and generally with good selectivity (>10-fold) against 164 kinases. Kinases with <10-fold selectivity were in the same family of kinases as CHK1/2, the CAM kinases, and some non-receptor tyrosine kinases (22). For LY2603618, CHK1 maintained >100-fold selectivity over the next target (PDK1) tested, and >1,500-fold selectivity over CHK2 (23). Dose–response curves for these inhibitors displayed >17-fold and >10-fold reductions in EC_50_ dependent on TSC2 status for LY2603618 and AZD7762, respectively (**Figures 1C, D**). Notably, TSC2-deficient cells treated with the dual CHK1/2 inhibitor, AZD7762 (**Figure 1D**), have a more potent and complete cellular EC_50_ when compared to the more selective CHK1 inhibitor, LY2693618 (**Figure 1C**), suggesting that CHK1/2 inhibition may be more effective at reducing the viability of TSC2-deficient cells. We note that AZD7762, while selective for CHK1/2, may be eliciting increased toxicity through off-target kinase engagement (22). Specifically, AZD7762 (EC_50_ = 37 nM) was 6-fold more potent for TSC2-deficient cells than LY2603618 (EC_50_ = 220 nM), and thus, we decided to pursue dual CHK1/2 inhibition in a TSC2-deficient setting.

**Figure 2:**
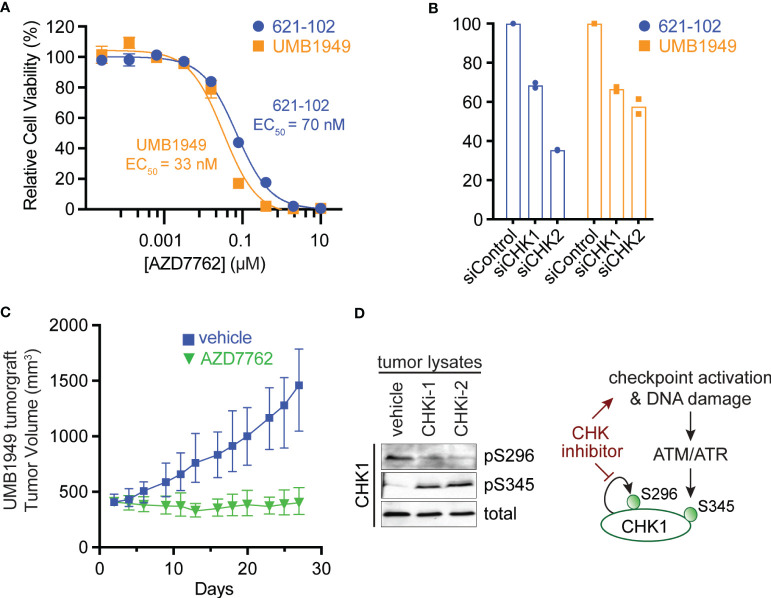
Antitumor efficacy of CHK1/2 inhibitors in patient-derived TSC2-deficient tumors. **(A)** Dose–response curves for AZD7762 in 621-102 or UMB1949 cell lines after 72 h of treatment. Data are presented as mean ± s.e.m. (n = 3) after normalization to control, and the best fit EC_50_ is indicated. **(B)** Relative viability measurements of 621-102 or UMB1949 cells 72 h after siRNA knockdown of either CHK1 or CHK2. **(C)** UMB1949 cell line tumorgraft tumor volume in NSG mice. After tumors reached 400 ± 25 mm^3^ in volume, mice were treated (i.p. injection) 5× per week with either vehicle (n = 9) or AZD7762 (12.5 mg/kg, n = 9); data presented as mean ± 95% confidence interval. **(D)** Western blot of tumor lysates from UMB1949 tumorgrafts treated with either vehicle or AZD7762. Lysates were probed for phospho-markers of CHK1 (pS296 and pS345) and total CHK1 used as loading control; CHKi-1 and CHKi-2 indicate two AZD7762-treated tumors from panel **(C)**.

To validate the primary chemical screen from 621-102 cells, we extended to a second patient-derived TSC cell line, UMB1949. We performed ten-point dose–response curves with AZD7762 in both TSC2-deficient cell lines, UMB1949 and 621-102. AZD7762 inhibited the growth of UMB1949 cells (EC_50_ = 33 nM) with a similar potency as 621-102 cells (**Figure 2A**). To validate that the inhibitory effects on viability were, in fact, due to CHK1 or CHK2 engagement, we used siRNA gene knockdown of CHK1 or CHK2 in the aforementioned cell lines (**Figure 2B**). While CHK1 knockdown resulted in a ~35% reduction in viability after 72 h, knockdown of CHK2 reduced viability up to 65%, consistent with increased potency of the dual CHK1/2 inhibitor AZD7762 relative to the CHK1-selective LY2603618. Given the role of TSC2 as a negative regulator of mTORC1 in response to various cellular stresses (24), including growth factor deprivation, we wanted to explore media conditions that allowed for cell growth and proliferation in a TSC2-deficient setting (**Supplemental Figure 1A**). In response to serum-restricted culture media (0.1% FBS), TSC2-deficient cells proliferated and responded to rapamycin treatment, while the TSC2-rescued cells were arrested and were unresponsive to rapamycin treatment (**Supplemental Figure 1B**) (25). Under these optimized growth conditions, we performed a secondary chemical screen in the TSC2-deficient 621-102 cells with a ten-point dose–response to acquire EC_50_ measurements (**Supplemental Table 2**). Notably, the rapalog everolimus was a potent inhibitor (**Supplemental Figure 1C**) as were both CHK inhibitors, LY2603618 and AZD7762 (**Supplemental Figure 1D**).

To expand the repertoire of potent compounds against TSC2-deficient cells, the top eighty-eight most potent (EC_50_) compounds with >60% reduction in viability were selected from the secondary chemical screen in 621-102 cells, as described above (**Supplemental Table 2**). Next, we performed a follow-up compound screen in both the 621-102 and UMB1949 cell lines with ten-point dose responses to report EC_50_ measurements (**Supplemental Table 3**). Under the limited serum and growth factor conditions, both CHK inhibitors were among the most potent compounds tested, maintaining EC_50_ < 7 nM for UMB1949 cells and EC_50_ < 3 nM for 621-102 (**Supplemental Figures 1D–F**). The general overview of the chemical screens performed is shown in **Supplemental Figure 1G**.

To test the effects of the dual CHK1/2 inhibitor, AZD7762, on tumor growth, we turned to *in vivo* mouse models. We first established a cell line tumorgraft model of the patient-derived UMB1949 in NSG (NOD *scid* gamma) mice. Next, we treated mice with either vehicle or AZD7762 (12.5 mg/kg, 5× weekly, for 28 days) and measured tumor volume (**Figure 2C**). AZD7762 treatment caused a significant reduction in UMB1949 TSC2-deficient tumor size compared with the tumor volume of the vehicle control. Strikingly, the growth delay, calculated at the days required to reach tumor volume of 500 mm^3^, was not achieved in the AZD7762 treatment group over the 28 days of treatment, while the control group reached 500 mm^3^ on day five. This indicates complete tumor stasis with CHK1/2 inhibition, as mice were enrolled to begin drug treatment when tumors reached 400 mm^3,^ and final tumor measurements did not reach starting tumor volumes with 0% tumor growth in the AZD7762-treated group. As expected, tumor lysates from AZD7762-treated mice showed decreased CHK1 serine 296 auto-phosphorylation (pS296), confirming *in vivo* CHK1 target inhibition (**Figure 2D**). In addition, increased CHK1 serine 345 phosphorylation (pS345) by ATM/ATR indicates strong checkpoint activation, replication stress, or DNA damage response by CHK1 inhibition (**Figure 2D**).

**Figure 3:**
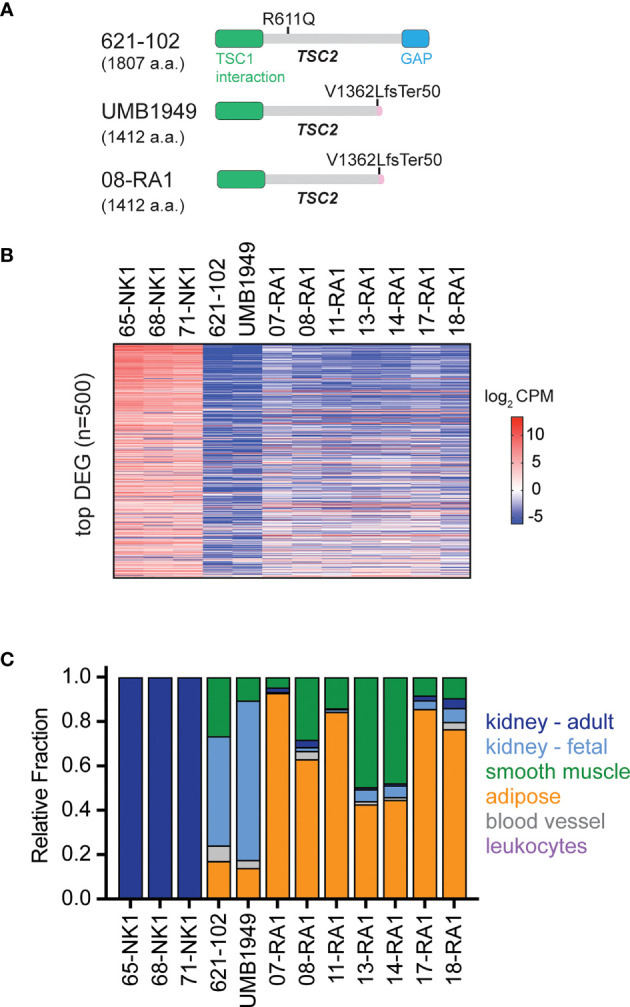
Molecular characterization of TSC cellular models. **(A)** Schematic for TSC2 protein structure in TSC2-deficient cell lines (621-102 and UMB1949) or 08-RA1 patient renal angiolipoma (RA) tumor; GAP, GTPase-activating protein. TSC2 deletion in patient-derived UMB1949 and renal angiomyolipoma 08-RA1. **(B)** Top 500 most differentially expressed genes (DEG), sorted high (top) to low (bottom) across the indicated samples and colored by log2 CPM according to the indicated scale. **(C)** The relative fraction of gene signatures estimated by CIBERSORT (gray, blood vessel; green, smooth muscle; orange, adipose; light blue, fetal kidney; dark blue, adult kidney; leukocytes purple).

The patient-derived UMB1949 cell line represents an additional cellular model of TSC with loss of TSC2 (**Figure 3A**), originally isolated from a male TSC patient with renal angiolipoma (18). Interestingly, we uncovered that UMB1949 cells reported by Lim et al. (18) were derived from a TSC patient that was part of our Martin et al. genomic profiling study (08-RA1) (3), which contained the same pathogenic frameshift deletion in TSC2 (**Figure 3A**). UMB1949 cell line identity matched to patient 08-RA1 was confirmed by rare variant analysis with minor allele frequency (MAF) < 0.01 in our exome data (3). To further establish a molecular understanding for the *in vitro* and *in vivo* models tested above, RNA-sequencing (**Supplemental Table 4**) was performed on UMB1949 and 621-102 cell lines and compared to our existing transcriptional landscape data for both normal kidney (NK) and RA tumors (3). When examining the top 500 differentially expressed genes (DEG) between normal kidneys and RA tumors, the transcriptional profiles of the two cell lines resembled the patient tumors (**Figure 3B**). In addition, cell-type deconvolution software (26) found adipose tissue and smooth muscle signatures shared between the cell models and patient angiomyolipomas, while expanded fetal kidney signatures were prominent in the cell lines (**Figure 3C**), perhaps due to the immortalization and effect, excess growth factors, or nutrients in cell culture media. Importantly, each cell line contained signatures of blood vessels, smooth muscle, and adipose tissue, the defining features of renal angiomyolipomas.

**Figure 4:**
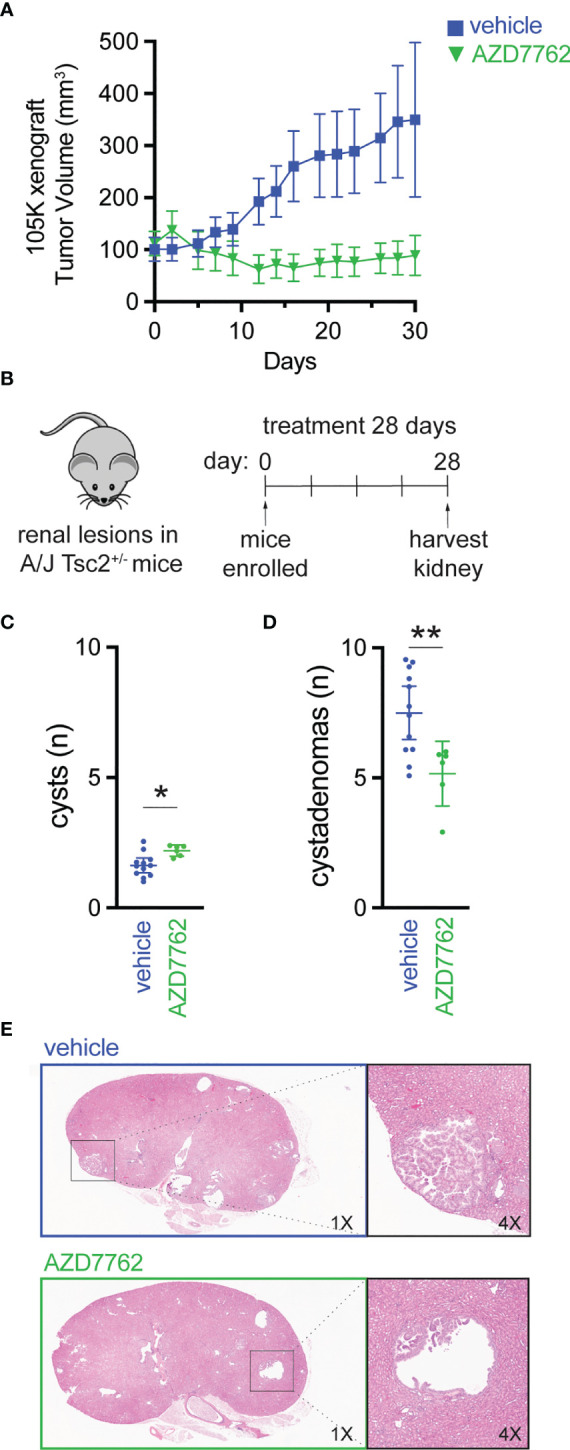
Checkpoint kinase inhibition as an effective antitumor strategy in preclinical tumor models of TSC2 loss. **(A)** Tumor volume of 105K xenografts in BALB/c nude mice treated once daily for 28 days with either vehicle (n = 14) or AZD7762 (12.5 mg/kg, n = 14); data presented as mean ± 95% confidence interval. **(B–D)** Eight-month-old A/J *Tsc2* ^+/-^ mice were treated daily with either vehicle (n = 10) or AZD7762 (12.5 mg/kg, n = 10) for 28 days; each treatment group had five male and five female mice. Data presented as mean ± 95% confidence interval and analyzed by one way ANOVA (*p < 0.05; **p < 0.01). **(E)** H&E-stained tissue of kidney sections from each indicated treatment group showing large cystadenoma lesion (vehicle) and cleared cyst (AZD7762).

We further evaluated CHK1/2 inhibition in a second mouse TSC model (27), in which 105K cells, a Tsc2-deficient cystadenoma cell line, derived from a renal tumor from a *Tsc2* ^+/-^ mouse (19). Tsc2-deficient 105K cells were engrafted subcutaneously into BALB/c-nu immunodeficient mice, and again, mice treated with AZD7762 had a significant reduction in tumor volume relative to vehicle-treated mice (**Figure 4A**). In addition, complete tumor stasis with CHK1/2 inhibition was again observed, as mice were enrolled to begin drug treatment when tumors reached 100 mm^3^ and final tumor measurements at 30 days did not reach starting tumor volumes. The AZD7762-treated mice had a >20% reduction in tumor volume as compared to the vehicle treated group with a >300% increase in tumor volume.

Next, we treated an A/J mouse model with heterozygous germline deletion of *Tsc2* (*A/J Tsc2^+/-^* mice) that spontaneously develops kidney pathology with age (20). In this model, tumors develop and progress from cysts to cystadenomas, and the severity of kidney cystadenomas increases with age. At 8 months of age, the age at which these mice have high tumor burden, *A/J Tsc2^+/-^* mice received AZD7762 or vehicle for 28 days (**Figure 4B**). After treatment, we harvested both left and right kidneys and performed histological analysis of cysts and cystadenomas to quantify overall cyst and tumor burden. Representative H&E-stained kidney sections used to quantify cysts and cystadenomas after treatment are shown (**Figure 4E**). Lesions were classified as cysts (0% < cell content < 25%) or cystadenomas (25% < cell content < 90%). AZD7762 treatment resulted in a 32% decrease in cystadenomas (**Figure 4D**) and a reduction in the progression from cyst to cystadenomas, as evidenced by the modest increase in the number of cysts (**Figure 4C**).

Here, we presented multiple chemical compound screens in TSC2-deficient cells as compared to TSC2-rescued cells. CHK inhibitors were potent inhibitors under all screening conditions tested, in particular in the restricted growth conditions that reveal the tumor-promoting properties of TSC2 loss. The antitumor potential of the dual CHK1/2 inhibitor, AZD7762, was demonstrated in three different animal models of TSC2-deficient tumors. The second-generation CHK inhibitor (prexasertib) is currently in clinical trials for various human cancers and appears to have mitigated toxicity concerns associated with the first-generation CHK inhibitor (28). Our data support further exploration of CHK inhibition in mTOR-driven pathologies and suggest a mechanistic connection between DNA damage response and mTOR signaling. Indeed, a growing body of evidence links mTORC1 activation to DNA damage repair (29–31). Consistent with these data, we previously reported a low mutational burden in TSC2-deficient patient tumors (3), suggesting efficient DNA repair mechanisms and perhaps a therapeutic vulnerability in tumors lacking functional TSC2.

## Data Availability Statement

The datasets presented in this study can be found in online repositories. The names of the repository/repositories and accession number(s) can be found in the article/**Supplementary Material**.

## Ethics Statement

The animal studies were reviewed and approved by Porsolt’s Ethical Committee and Van Andel Institute Review Committee.

## Author Contributions

Designing research studies: KM., JM, DA. Conducting experiments: KM, JK, KS, C-YC, NS, MB, MK. Acquiring data: KM, RV, JM. Analyzing data: KM, RV, NS, JM, DA. Providing reagents: DA. Writing the manuscript: RV, KM, JM. Acquisition of funding: RV, JM, DA. All authors contributed to the article and approved the submitted version.

## Funding

RV (K00CA245821) and JM (R21CA263133) have research support from the National Cancer Institute. This work was supported by grants and funding from the Michigan Strategic Fund, Tuberous Sclerosis Complex Alliance, Blue Cross Blue Shield of Michigan Foundation, and individual donors.

## Conflict of Interest

JM has consulting agreements with Merck, research support from Erasca, and scholarly activity and support from Translational Genomics Research Institute (non-profit organization). JK’s current affiliation is with AbbVie.

The remaining authors declare that the research was conducted in the absence of any commercial or financial relationships that could be construed as a potential conflict of interest.

## Publisher’s Note

All claims expressed in this article are solely those of the authors and do not necessarily represent those of their affiliated organizations, or those of the publisher, the editors and the reviewers. Any product that may be evaluated in this article, or claim that may be made by its manufacturer, is not guaranteed or endorsed by the publisher.
