# Supplementary material for: Chemical Biology Screening Identifies a Vulnerability to Checkpoint Kinase Inhibitors in TSC2-Deficient Renal Angiomyolipomas
Source: Front Oncol. 2022 Mar 10;12:852859. doi: 10.3389/fonc.2022.852859 (PMC8960247; doi:10.3389/fonc.2022.852859)
Supplement: Supplementary file 1 [file DataSheet_1.pdf]

Supplemental Figure 1. (**A**) Illustration of nutrient and growth factor signaling through TSC2 and mTORC1. (**B**) Viability measurements of TSC2-deficient (621-102) or TSC2-rescued (621-103) cells in limited growth factor conditions (DMEM + 0.1% FBS) treated with either vehicle (DMSO) or rapamycin (50 nM), data presented as mean ± s.e.m. (n=5). (**C**) Everolimus EC_{50} measurements in TSC2-deficient (621-102) cells in the limited growth conditions. (**D**) Scatter plot of EC_{50} measurements of relative viability in TSC2-deficient cells, 621-102 versus UMB1949, in the limited growth conditions (Supplemental Table 2). Dose response curves for the indicated CHK inhibitors LY2603628 (**E**) and AZD7762 (**F**) in 621-102 cells (blue) or UMB1949 cells (orange). (**G**) General details and workflow for the three compound screens performed in this manuscript.

Supplemental Table 1 – Relative viability measurements from CellTiter Glo data for the primary chemical screen in 621-102 (TSC2-deficient) and 621-103 (TSC2-rescued) in 10% FBS containing media.

Supplemental Table 2 – Relative viability measurements from CellTiter Glo data for the secondary screen in 621-102 cells in 0.1% FBS containing media.

Supplemental Table 3 – Relative viability measurements from CellTiter Glo data for follow-up screen in 621-102 and UMB1949 cells in 0.1% FBS containing media.

Supplemental Table 4 – RNA-sequencing data used in this study presented in log_2 counts per million mapped reads (CPM). Normal kidney (NK) and renal angiomyolipoma (RA) samples are from Martin *et al.* (Nat. Commun., 2017).
