# Supplementary figures and images for: Chemical Biology Screening Identifies a Vulnerability to Checkpoint Kinase Inhibitors in TSC2-Deficient Renal Angiomyolipomas
Source: Front Oncol. 2022 Mar 10;12:852859. doi: 10.3389/fonc.2022.852859 (PMC8960247; doi:10.3389/fonc.2022.852859)

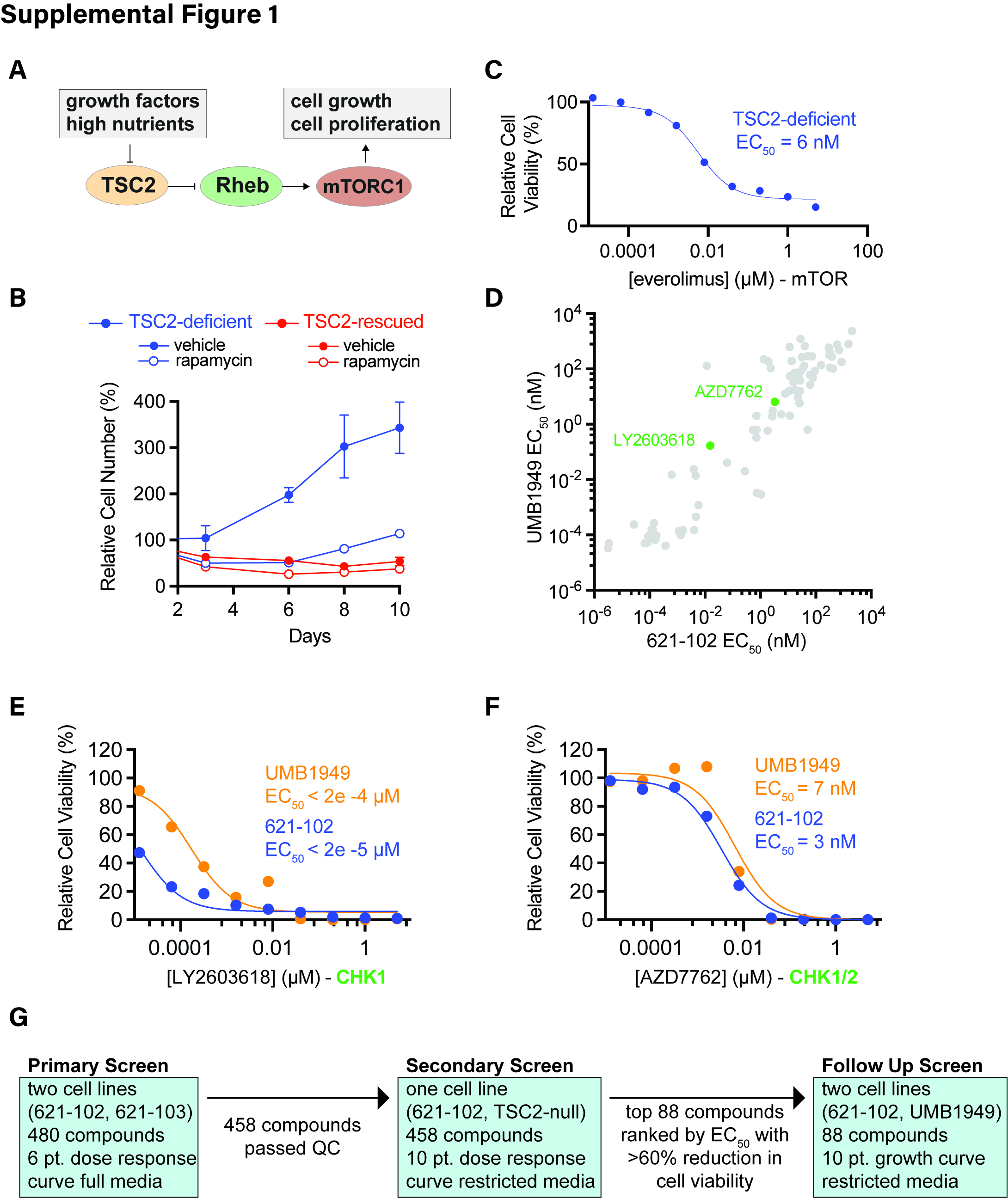

Supplement: Supplementary file 2 [file Image_1.tif]
